# Steroid-mediated liver steatosis is CD1d-dependent, while steroid-induced liver necrosis, inflammation, and metabolic changes are CD1d-independent

**DOI:** 10.1186/s12876-022-02242-9

**Published:** 2022-04-07

**Authors:** Tomer Adar, Ami Ben Ya’acov, Yehudit Shabat, Meir Mizrahi, Lida Zolotarov, Yoav Lichtenstein, Yaron Ilan

**Affiliations:** 1 Faculty of Medicine, Department of Medicine, Hadassah Medical Center, Hebrew University, POB 1200, 91120 Jerusalem, Israel; 2 Digestive Disease Institute, Shaare-Zedek Medical Center, Jerusalem, Israel

**Keywords:** Steroids, NKT cells, NAFLD, NASH, Glycosphingolipids, Glucocerebroside

## Abstract

**Introduction:**

Glucocorticoids contribute to the pathogenesis of non-alcoholic fatty liver disease (NAFLD). Natural killer T cells play a role in the pathogenesis of NAFLD and response to steroids. The present study aimed to determine the role of CD1d in steroid-mediated metabolic derangement and the steroid-protective effect of glycosphingolipids.

**Methods:**

Ten groups of mice were studied. Steroids were orally administered to C57BL/6 mice to assess the therapeutic effect of β-glucosylceramide (GC) on the development of steroid-mediated liver damage and metabolic derangements. The role of CD1d in the pathogenesis of steroid-induced liver damage and in mediating the hepatoprotective effect of GC was studied in CD1d^−/−^ mice.

**Results:**

A model of oral administration of steroids was established, resulting in insulin resistance, hyperinsulinemia, hypertriglyceridemia, liver steatosis, and hepatocellular injury. Steroid administration to CD1d^−/−^ mice was associated with hyperglycemia and hypertriglyceridemia. However, CD1d^−/−^ mice did not manifest marked steroid-induced steatosis. GC treatment alleviated steroid-associated metabolic derangements and liver injury independent of CD1d expression.

**Conclusion:**

A steroid-mediated model of NAFLD and metabolic derangements was established in which steroid-mediated steatosis was CD1d-dependent while steroid-induced liver necrosis, inflammation, and metabolic changes were CD1d-independent, which may support a dichotomy between steatosis and steatohepatitis in NAFLD.

**Supplementary Information:**

The online version contains supplementary material available at 10.1186/s12876-022-02242-9.

## Significance statement

The paper describes a steroid-mediated model of NAFLD and metabolic derangements. Steroid-mediated steatosis was CD1d-dependent, while steroidinduced liver necrosis, inflammation, and metabolic changes were CD1d-independent.

## Introduction

Glucocorticoid administration inhibits mitochondrial beta-oxidation and lipid beta-peroxidation enzymes, leading to hepatic steatosis. Glucocorticoids inhibit medium- and short-chain acyl-CoA dehydrogenation and hepatic lipid secretion [1]. In addition, corticosteroids promote de novo fatty acid synthesis by stimulating lipogenic enzymes, including fatty acid synthase, acetyl-CoA carboxylase, and 11 beta-hydroxysteroids dehydrogenase type 1 in the liver [2]. Glucocorticoids increase hepatic phosphatidate phosphatase (PAP1) activity, promoting fatty acid storage as triacylglycerols (TAGs). TAGs serve for beta-oxidation of very-low-density lipoprotein (VLDL) secretion [3].

Glucocorticoids are catabolic, leading to energy substrates during stress [4, 5]. Steroids play a role in developing diet-induced obesity by modulating fasting triglyceride metabolism [6]. During the fasting state, glucocorticoids mobilize lipids, thus increasing fatty acid delivery, and in the fed state, they promote lipid accumulation [7]. Steroids augment the levels of circulating fatty acids by increasing the caloric and dietary fat intake and the hydrolysis of circulating triglycerides by lipoprotein lipase activity [4]. The process may be associated with ectopic fat distribution in the liver, muscle, and central adipocytes.

Extreme action of cortisol and fatty acids contributes to insulin resistance and associated pathologies [8]. An imbalance of the hypothalamic–pituitary–adrenal axis exacerbates the effects of cortisol [8]. Fatty acids and cortisol induce insulin resistance, augmenting lipogenesis in the liver and hepatic secretion of glucose and VLDL [9]. In high insulin concentrations, cortisol promotes energy deposition and leads to obesity [8]. Lowered glucose metabolism is associated with altered fatty acid supply, composition, and utilization, as well as with "lipotoxicity," which further contributes to steroid-mediated liver damage [10].

Glucocorticoids may play a role in the pathogenesis of all stages of non-alcoholic fatty liver disease (NAFLD) by affecting both the liver and the adipose tissue [7]. Both microvesicular steatosis and ultrastructural mitochondrial lesions have been reported [1]. Chronic glucocorticoids and a high-fat diet (HFD) independently induce insulin resistance, abdominal obesity, and NAFLD. In a rodent model, the combination of glucocorticoids and an HFD resulted in central obesity, insulin resistance, increased liver lipid content, and fibrosis [11]. The liver damage was independent of inflammation [11] and the anti-inflammatory effect of steroids [12].

Intravenous glucocorticoids as a treatment for various steroid-responsive diseases may be associated with liver toxicity. An increase in anabolic–androgenic steroid-mediated hepatotoxicity is a significant public health problem [13]. Both hepatocellular and cholestatic features were described. Statins, for example, when concomitantly employed with methylprednisolone, lead to liver dysfunction [14]. Natural killer T (NKT) cells are a unique subset mostly abundant in murine and human livers. NKT cells are divided into type 1 and type 2 according to their dependence on the interaction with CD1d, a non-polymorphic glycolipid antigen-presenting molecule. NKT cells modulate hepatic inflammation through CD1d recognition in conjunction with glycolipid antigen, and they have a protective role in the pathogenesis of NAFLD and the response to steroids [15–17]. Glycosphingolipids (GSLs) exert a hepatoprotective effect in animal models by alleviating liver inflammation via an NKT cell-mediated effect [18–28]. The present study aimed to determine the role of CD1d in steroid-mediated liver injury, metabolic derangement, and the steroid-protective effect of GSLs.

## Materials and methods

### Animals

10-week-old male C57BL/6 mice were purchased from Harlan Laboratories (Jerusalem, Israel). CD1d^−/−^ mice (C57 background) were kindly provided by Dr. Luc Van Kaer, Department of Microbiology and Immunology Medical Center North, Vanderbilt University School of Medicine, Nashville, TN. Mice were maintained in the animal core of the Hadassah-Hebrew University Medical School. All mice were administered standard laboratory chow and water ad libitum and maintained a 12-h light/dark cycle. The animal experiments were carried out according to the guidelines of the Hebrew University-Hadassah Institutional Committee for Care and Use of Laboratory Animals and with the committee's approval.

### Induction of liver and metabolic damage and GSL treatment

Metabolic and liver damage were induced by oral administration of Dexamethasone (0.5 mg/day). β-glucosylceramide (GC) was purchased from Avanti Polar Lipids (Alabaster, AL, USA), dissolved in a vehicle of a mixture of 30% Cremophor EL (Sigma, Rehovot, Israel) and ethanol (1:1) in PBS, and administered orally at a dose of 15 µg/day.

### Study design

Mice in all groups were followed to determine the effect of steroids and GC on metabolic derangements and liver damage. The graphs are representative of three separate experiments in which similar results were obtained.

#### Experimental groups

Ten groups of mice were studied in three experiments (Table 1). In the first part of the study, three groups of C57BL/6 mice were followed for eight weeks to assess the preventive effect of GC on the development of steroid-mediated liver damage. Group A received vehicle alone, Group B received Dexamethasone, and group C received a combination of Dexamethasone and GC. To determine the therapeutic effect of GC on the existing damage, four groups of C57BL/6 mice were studied in the second part of the study.

**Table 1:** Experimental groups

Group	Mice	Treatment	Duration (weeks)
A	C57BL/6	Vehicle	8
B	C57BL/6	Dexamethasone	8
C	C57BL/6	Dexamethasone + β-glucosylceramide	8
D	C57BL/6	Vehicle	12
E	C57BL/6	Dexamethasone	12
F	C57BL/6	β-glucosylceramide	12
G	C57BL/6	Dexamethasone for six weeks followed by Dexamethasone + β-glucosylceramide additional two weeks	Six weeks for induction and two weeks for treatment
H	CD1d-/-	Vehicle	8
I	CD1d-/-	Dexamethasone	8
J	CD1d-/-	Dexamethasone + β-glucosylceramide	8

Mice in group D received vehicle alone; group E received Dexamethasone, group F received a combination of Dexamethasone and GC, mice in group G received Dexamethasone for six weeks followed by two weeks of administration of Dexamethasone in combination with GC. To determine the involvement of CD1d in steroid-induced liver damage and the steroid-protective effect of GC, we used CD1d^−/−^ mice were for the third part of the study: Mice in group H received vehicle alone, group I received Dexamethasone, and group J received a combination of Dexamethasone and GC for eight weeks.

### Fasting serum glucose and insulin levels

Fasting serum glucose levels were measured using a standard glucometer. The manufacturer's instructions determined serum insulin levels using a commercially available ELISA kit (Mercodia AB; Uppsala, Sweden).

### Glucose tolerance test (GTT)

A glucose tolerance test (GTT) was performed using a standard glucometer by oral glucose administration (1.25 g/kg) with repeated glucose level tests (the tail vein using a standard glucometer.

### Liver enzymes and triglyceride levels

Serum aspartyl transaminase (AST), alanine aminotransferase (ALT), and serum triglycerides were measured using commercially available sticks by a Reflovet Plus analyzer (Roche Diagnostics, GmbH, Mannheim, Germany).

### Histological examination

A portion of each excised liver from all mice in all groups was formalin-fixed. Tissue samples were embedded in paraffin, and 5-μm sections were cut. Sections were stained with hematoxylin and eosin (H&E) and read by a blinded pathologist.

### Hepatic triglycerides content

Accumulation of intrahepatic triglycerides (TGs) was quantified using a modification of the Folch method [29]. Triglycerides were extracted by a GPO-Trinder kit (Sigma, Rehovot, Israel), then quantified by a spectrophotometer, and normalized levels to the homogenate protein content.

### Serum cytokine levels

Serum levels of IL10 and IL17 were measured using a commercially available sandwich ELISA kit (Quantikine, R&D Systems, MN, USA).

### Statistical analysis

Statistical analysis was performed using the Anova test. A *p-*value less than 0.05 was considered significant.

## Results

### Steroid treatment-induced insulin resistance, hyperlipidemia, and liver damage

**Fig. 1:**
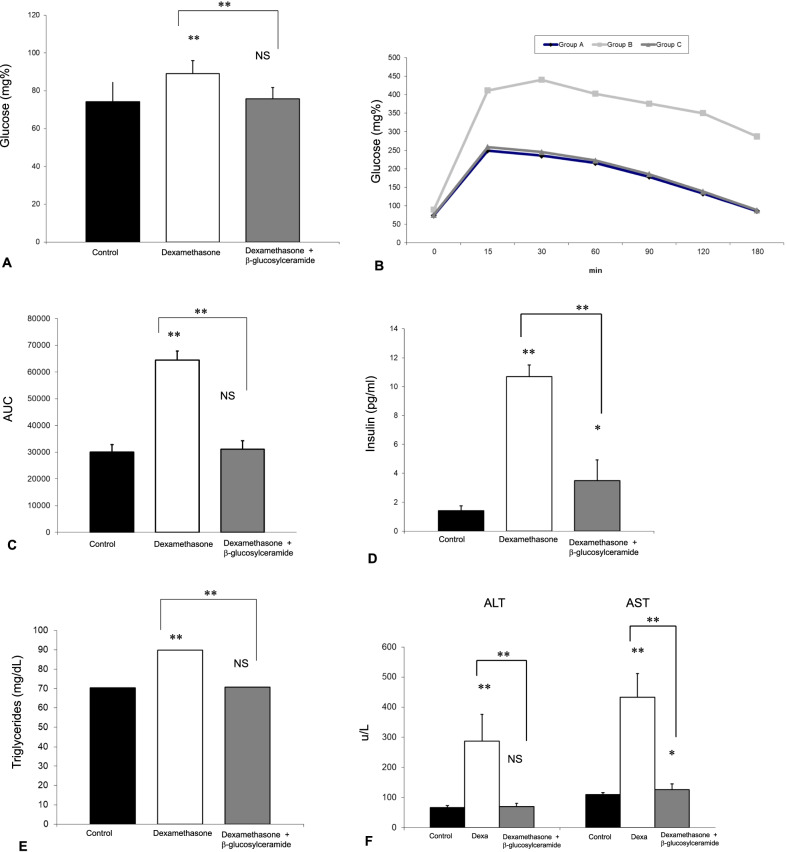
Effect of steroid treatment on metabolic and liver parameters. Three groups of C57BL/6 mice were followed for eight weeks to assess the preventive effect of GC on the development of steroid-mediated liver damage. Group A received vehicle alone; Group B received Dexamethasone, and group C received a combination of Dexamethasone with GC. Mice were followed for fasting serum glucose levels (**a**); postprandial glucose levels as manifested by glucose tolerance test absolute values (**b**) and AUC (**c**); serum insulin levels (**d**); serum triglyceride levels (**e**); and AST and ALT liver enzymes (**f**) (**p < 0.01, *p < 0.05).

Figure 1 shows the effect of steroids on metabolic parameters and liver damage. Oral administration of Dexamethasone resulted in insulin resistance manifested by increased fasting serum glucose levels (Fig. 1a). It increased GTT as shown by the study curves (Fig. 1b), by the calculated areas under the curve (AUC) (Fig. 1c), and by increased serum insulin levels (Fig. 1d). Steroid treatment also increased serum triglyceride levels (Fig. 1e). The effect of steroids on insulin and lipid metabolism was associated with a significant hepatocellular injury manifested by an increase in ALT and AST serum levels (Fig. 1f).

### Oral administration of β-Glucosylceramide prevented the steroid-mediated metabolic and liver damage

Co-administration of GC and steroids prevented the increase in fasting glucose levels (Fig. 1a), postprandial hyperglycemia (Fig. 1b, c), hyperinsulinemia (Fig. 1d), hypertriglyceridemia (Fig. 1e), and the increase in liver enzymes (Fig. 1f), signifying its protective effect against steroid-mediated metabolic and hepatic damage.

### Oral administration of β-Glucosylceramide alleviated steroid-mediated metabolic and liver damage

**Fig. 2:**
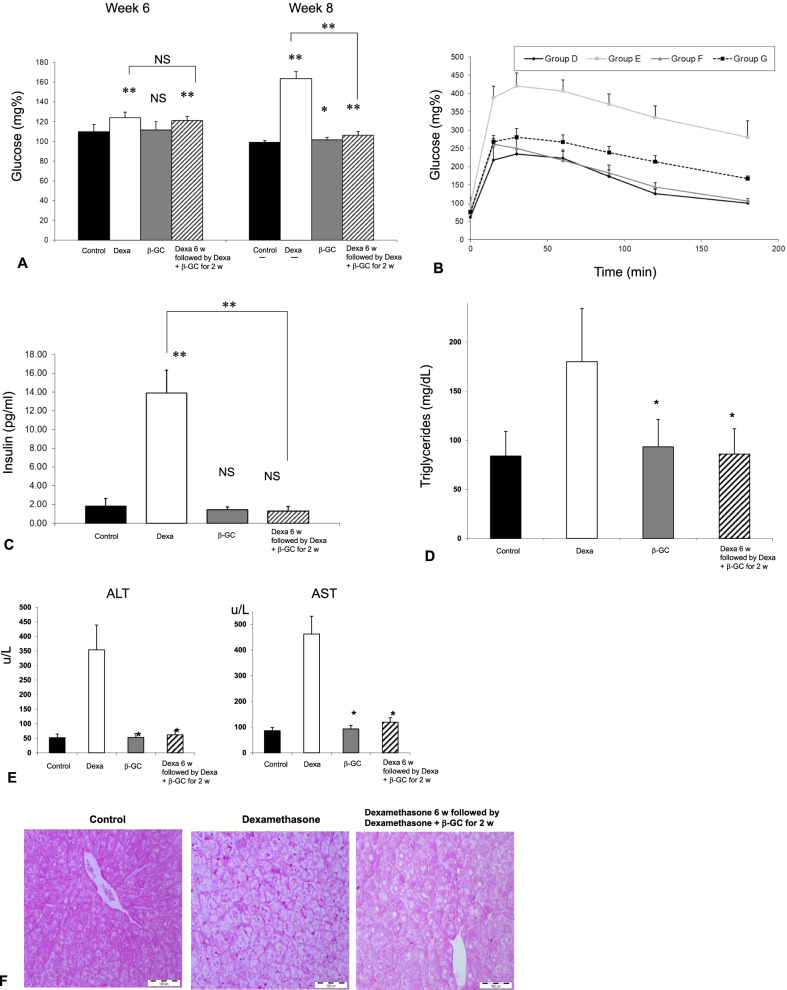

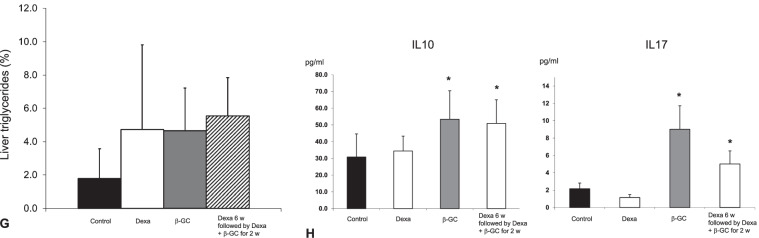
The therapeutic effect of GC on existing metabolic and liver damage was studied in four groups of C57BL/6 mice. Mice in group D received the vehicle alone; Group E received Dexamethasone; Group F received a combination of Dexamethasone with GC; mice in group G received Dexamethasone for six weeks followed by two weeks of Dexamethasone with GC. Mice were followed for fasting glucose levels **(a)**; postprandial glucose levels as manifested by glucose tolerance test **(b)**; serum insulin levels **(c)**; serum triglyceride levels **(d)**; AST and ALT liver enzymes **(e)**; liver histology (H&E X10) **(f)**; hepatic triglycerides content **(g)**; and for serum levels of IL10 and IL17 **(h)** (**p < 0.01, *p < 0.05)

The second part of the study (Fig. 2) aimed to determine the therapeutic effect of orally administering GC following steroid-mediated metabolic and liver damage induction. GC therapy was associated with a reduction of fasting plasma glucose levels on week eight (Fig. 2a), improvement in the GTT (Fig. 2b), and reduction of serum insulin levels (Fig. 2c). These effects were comparable to the protective effect of GC when administered for prevention (group F vs. group G, prevention vs. treatment, respectively). Similarly, GC therapy alleviated steroid-mediated hypertriglyceridemia (Fig. 2d) and liver injury by reducing ALT and AST serum levels (Fig. 2e). Histological analysis showed a reduction in ballooning and cell apoptosis (Fig. 2f). However, GC did not affect total liver triglyceride content in WT mice (Fig. 2g). GC therapy was associated with an increase in IL10 and IL17 serum levels, suggesting an effect on the systemic immune milieu in this system (Fig. 2h).

### CD1d-dependence of liver steatosis and CD1d-independence of the steroid-mediated damage and therapeutic effect of GC

**Fig. 3:**
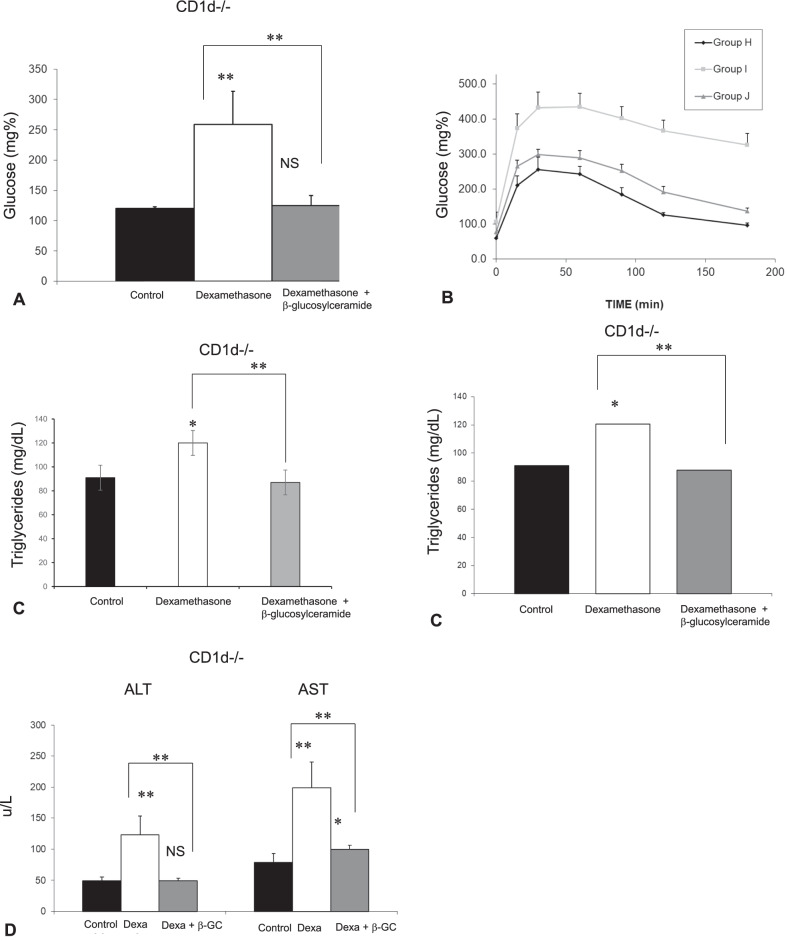

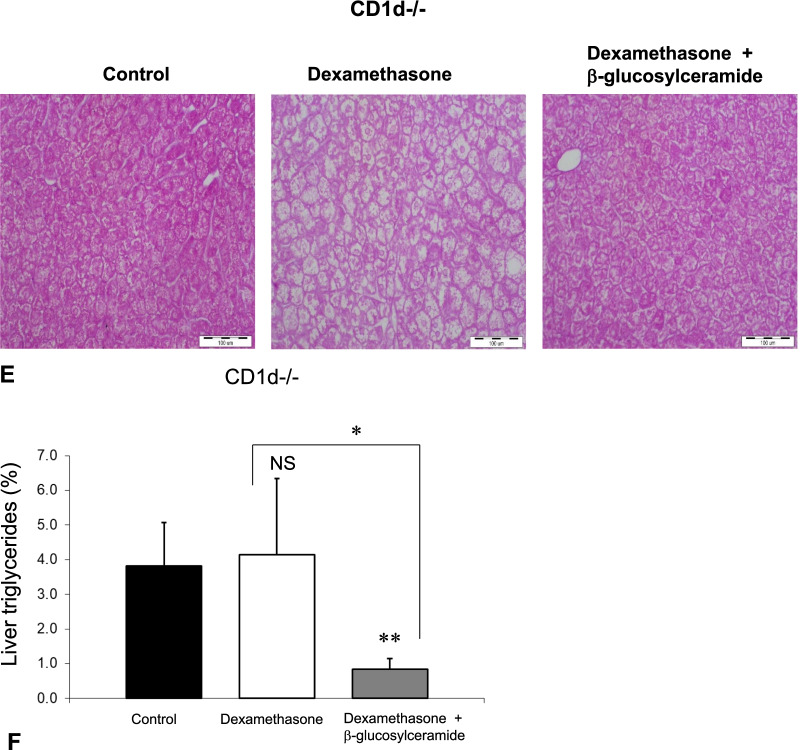
The CD1d-dependency of the steroid-induced liver damage, and the steroid-protective effect of GC, were studied in three groups of CD1d^−/−^ mice. Mice in group H received the vehicle alone; Group I received Dexamethasone, and Group J received a combination of Dexamethasone with GC for eight weeks. Mice were followed for fasting glucose levels **(a)**; postprandial glucose levels as manifested by glucose tolerance test **(b)**; serum triglyceride levels **(c)**; AST and ALT liver enzymes **(d)**; liver histology (H&E X10) **(e)**; and hepatic triglycerides content **(f)** (**p < 0.01, *p < 0.05)

The third part of the study (Fig. 3) aimed to determine the involvement of NKT cells in steroid-mediated damage and the therapeutic effect of GC. Steroid administration in CD1d^−/−^ mice was associated with significant insulin resistance manifested by increased fasting serum glucose levels (Fig. 3a) and increased GTT (Fig. 3b), along with hypertriglyceridemia (Fig. 3c). Administration of steroids to CD1d^−/−^ mice was associated with significant liver damage manifested by increased liver enzymes (Fig. 3d) and necrosis as seen by histological examination (Fig. 3e). However, CD1d^−/−^ mice manifested the absence of an increase in liver TG content following steroid administration (Fig. 3f).

The protective effect of GC was independent of CD1d. GC treatment alleviated steroid-mediated hyperglycemia (Fig. 3a, b) and hypertriglyceridemia (Fig. 3c). GC treatment prevented steroid-mediated liver damage as manifested by decreased liver enzymes (Fig. 3d). Histologically, GC treatment alleviated steroid-mediated hepatocyte ballooning, cell apoptosis, and steatosis. Interestingly, GC treatment significantly reduced the triglyceride content (Fig. 3e), an effect that was not observed in WT mice.

Data of all experiments are included in the Additional file 1.

## Discussion

The present study established a model of oral steroid administration for eight weeks to simulate insulin resistance and the associated liver injury similar to that noted in patients with metabolic syndrome. Steroid-treated mice exhibited insulin resistance, hypertriglyceridemia, and hepatocellular injury. These results support previously published data on the effect of steroids on the liver [7, 11]. CD1d^−/−^ mice were used for determining the potential role of CD1d in mediating steroid damage. The steroid-associated hyperglycemia and hypertriglyceridemia were independent of CD1d. However, CD1d^−/−^ mice did not manifest marked steroid-induced steatosis. The relative resistance to steatosis was independent of the steroid-induced liver damage in these mice manifested by the increased liver enzymes and hepatocytes necrosis on histology. The present study supports a role for CD1d in the development of steatosis per se while not preventing steroid-mediated hepatocyte damage or metabolic derangements. These results suggest a partial role in activating CD1d-positive cells in NAFLD development.

Hepatic steatosis is associated with activation of the innate immune system [30]. Leptin-deficient *ob/ob* mice exhibit metabolic and inflammatory features that mimic human NAFLD [31–34]. These mice exhibit a decrease in liver NKT cells [35, 36], suggesting that these lymphocytes may regulate the development of NAFLD [30]. In this model, leptin deficiency modulates inflammation independently of hepatic steatosis. An obese/steatosis HFD model, associated with weight gain and hepatic steatosis, is also associated with a reduction in liver NKT cells [30, 37, 38]. These findings can be interpreted as a lack of a full protective effect of NKT deficiency, wherein a decrease of NKT cells is associated with more significant damage, independent of steatosis. These observations may also result from post-activation-mediated cell death, wherein NKT activation leads to greater steatohepatitis and a subsequent decrease in NKT cells [27]. Endogenous IL-12 released by Kupffer cells (KC) in obesity may promote the release of IFN-γ by activated NKT cells, with a subsequent decrease of intrahepatic NKTs [30, 39]. Dietary fatty acids are linked to hepatic NKT cell deficiency [40], showing that dietary saturated fatty acids (SFA) or monounsaturated fatty acids (MUFA), but not polyunsaturated fatty acids (PUFA), deplete liver NKT lymphocytes [30, 40].

Obesity is characterized by low-grade systemic inflammation, and the adipocyte-macrophage interaction contributes to its development [41, 42]. An interplay between insulin resistance and inflammation contributes to hepatic steatosis and steatohepatitis. Anti-inflammatory agents are beneficial in animal models and patients with NASH [25, 43]. The present study results support the lack of a direct relationship between steroid-induced metabolic changes and hepatocyte injury and steatosis, implying a pathogenesis dichotomy between steatosis and liver damage in NAFLD. NAFLD comprises a spectrum from pure steatosis to NASH. The present data suggest that the mechanism that induces liver damage in NASH may be independent of steatosis and can further explain the liver damage in lean NASH patients and the fact that the majority of patients with simple steatosis do not develop NASH [44, 45].

GC acts as liver-protecting agents and may prevent drug-induced liver injury (DILI) of several hepatotoxic drugs [18]. In the present study, administering GC prevented and treated the steroid-mediated liver damage as observed by a reduction of ALT and AST serum levels and a decrease in the levels of ballooning and apoptosis upon histological examination. GC alleviated steroid-induced hyperglycemia, hyperinsulinemia, and hypertriglyceridemia. The protective effect of GC was independent of CD1d. However, while CD1d^−/−^ mice showed less steroid-mediated steatosis, GC treatment exerted a more profound effect on reducing steatosis in these mice compared with WT mice. These data support a dichotomy in GC function for reducing steroid-mediated steatosis, which is prevented by CD1d, which was not seen for the effect of GC on hepatocellular damage.

Manipulations of NKT cells are beneficial in the settings of diabetes, fatty liver disease, and NASH in animal models and humans [16–20, 23, 39, 40, 46–50]. GC treatment exerts a fat-reducing effect in animal models and humans [23, 33]. The present data support CD1d^+^ lymphocyte plasticity, which underlines the differences noted between the different models. NKT cells were shown to be either beneficial or associated with liver damage under different conditions in an environment- and disease-dependent manner [47, 48]. GC treatment alleviates immunologically incongruous disorders, suggesting it is associated with a "fine-tuning" of immune responses by altering NKT-plasticity [48, 49], and this effect may be CD1d-mediated [50, 51]. The CD1d-independence of the GC-fat reduction effect under steroid therapy in the present study may also suggest an alternative CD1d-independent liver protective effect of GC. A CD1d evading mechanism similar to the one described for infectious agents may also play a role under these conditions [52]. Interleukin-1 is an essential target of steroid therapy [53] and promotes the inflammation in NASH [51]; a possible link via IL1 and GC may explain these findings.

GC therapy has been associated with increased IL10 and IL17 serum levels, suggesting its effect on the systemic immune system. Serum IL-10 levels are significantly lower in patients with NASH [54], while IL-17 exacerbates hepatic steatosis and inflammation in NAFLD [55]. As only two cytokines were measured in the present study, a general alteration of the cytokine milieu in an anti-inflammatory direction may underlie the noted beneficial clinical effect. Future studies will need to dissect the subsets of cells mediating these effects.

The study's limitation is the lack of analysis of phenotypes of wild type and CD1d-knockout mice side by side, by recruiting both wild type and knockout littermates, instead of performing all the analyses separately. Additional tests to determine the effect of therapies on lipid accumulation in the liver will be conducted in future studies.

## Conclusion

Our study established a model of NAFLD and metabolic derangements by steroid administration for eight weeks in which the steroid-mediated steatosis was CD1d-dependent while steroid-induced liver necrosis and inflammation, insulin resistance, and hyperlipidemia were CD1d-independent. The results may support a pathogenesis dichotomy between steatosis and steatohepatitis in NAFLD. CD1d^−/−^ mice were relatively resistant to steroid-induced steatosis, suggesting that NKT cells play a role in fat accumulation. The data may support the lack of a direct association between steatosis per se and liver damage. The beneficial effect of GC to alleviate the steroid induced-metabolic changes and liver damage were independent of CD1d, while CD1d prevented the ability of GC to reduce steroid-mediated steatosis. Overall, the data support the potential use of GC as both a liver protector with steroid therapy and as a drug to treat NAFLD. Our data also support the need for combination therapy in patients with NASH wherein both inflammatory and metabolic pathways need to be targeted [56].

## Supplementary Information

**Additional file 1**. Data of all experiments.

## Data Availability

All data of is included in the supplementary file.

## Abbreviations

NAFLD: Non-alcoholic fatty liver disease
GSLs: Glycosphingolipids
NKT: Natural killer T cells
PAP1: Phosphatidate phosphatase
TAGs: Triacylglycerols
HFD: High-fat diet
GC: β-Glucosylceramide
GTT: Glucose tolerance test
AST: Aspartyl transaminase
ALT: Alanine aminotransferase
TGs: Triglycerides
NASH: Non-alcoholic steatohepatitis
KC: Kupffer cells
IFN-γ: Interferon-gamma
SFA: Saturated fatty acids
MUFA: Monounsaturated fatty acids
PUFA: Polyunsaturated fatty acids
DILI: Drug-induced liver injury
